# A review of machine learning in toxicology: current practices and reporting gaps

**DOI:** 10.1007/s00204-026-04393-0

**Published:** 2026-04-30

**Authors:** Franziska Kappenberg, Marieke Stolte, Luca Sauer, Julia C. Duda, Michael Lau, Leonie Schürmeyer, Huiying Zhou, Holger Schwender, Kirsten Schorning, Jörg Rahnenführer

**Affiliations:** 1 Department of Statistics, TU Dortmund University, Vogelpothsweg 87, Dortmund, 44227 Germany; 2 Mathematical Institute, Heinrich Heine University, Universitätsstrasse 1, Düsseldorf, 40225 Germany; 3 Institute for Medical Biometry, Informatics and Epidemiology, University of Bonn, University Hospital Bonn, Venusberg-Campus 1, Bonn, 53127 Germany; 4 eBay Inc., 2025 Hamilton Avenue, San José, 95125 California USA

**Keywords:** Review, Machine Learning, Artificial Intelligence, Toxicology, QSAR, Reporting

## Abstract

In recent years, machine learning and artificial intelligence approaches have been increasingly applied in the context of toxicological risk assessment. Many published overview, review, and comment papers discuss advantages, disadvantages, success stories, and open challenges for the application of machine learning models in toxicology. Machine learning methods using information from in vitro experiments can help to avoid animal experiments, thus allowing for larger numbers of experiments to be conducted. Drawbacks of machine learning models are the lack of mechanistic interpretability and the need for large amounts of high-quality data. In this work, we present a literature review of papers indexed in PubMed or published in the journal Computational Toxicology in the years 2022 to 2024, to assess the usage of machine learning methods in toxicology as well as the practices in reporting of methods and corresponding results. We do not address the suitability or the performance of methods, which is impossible to assess objectively without reanalysis on raw data, but focus on common practices and gaps in reporting. Major results are that many different machine learning methods are used in toxicology, often with appropriate internal validation. However, in only half of the cases, interpretation methods are used to address the problem that these models often make predictions as a black box. Moreover, there are very frequent gaps in reporting, in particular related to handling of missing values, and availability of data and code. Thus, this review can serve as a starting point for further tailored methodological research and guidance.

**Supplementary Information:**

The online version contains supplementary material available at 10.1007/s00204-026-04393-0.

## Introduction – State of the art of machine learning in toxicology

### Research areas in toxicology

Humans are exposed to a multitude of substances at any given time. This includes substances in food and in drugs, as well as substances one is exposed to via the environment, e.g. pesticides, or during work (occupational exposures). Thus, toxicity assessment of substances is of great interest. To assess how a potential drug interacts with the human body, the aspects of Absorption, Distribution, Metabolism, Excretion, and sometimes the Toxicity of substances are considered. These attributes are summarized as ADME(T), and their characterization is at the core of pharmacological research (Gombar et al. 2003).

Assessing the bioactivity, e.g. the toxicity, of substances, is a topic of relevance in several application areas, with drug development being one of the most important areas. For the pre-clinical phases of drug development, animal-based in vivo experiments are becoming less common, following the 3Rs (replacement, reduction, refinement) principle. Instead, so-called new approach methodologies (NAM) are becoming more popular (Sewell et al. 2024). NAM is an umbrella term for any kind of approach or method, e.g. in vitro or in silico, that yields insight into risk assessment of substances and avoids animal testing.

Nevertheless, for a more complete shift from animal-based testing to NAM approaches in the context of risk assessments, technical, cultural, and regulatory barriers must be overcome. Sewell et al. (2024) give an overview of the challenges with respect to the different types of barriers, but also give examples of specific applications where NAMs have successfully been applied alongside or instead of animal testing. The U.S. Food and Drug Administration (FDA) has recently published a roadmap to reducing animal testing by using NAMs, and thus improving the ethical and economic aspects of drug development, where ML and AI predictive models are explicitly listed among the available in silico tools (U.S. Food and Drug Administration 2025). They give specific steps for an implementation of reduced animal testing in the next 3 years, and steps for validation of NAMs and of integration of NAMs into regulatory processes. However, a recent work by Gerke et al. (2026) raises concerns about the optimistic time-frame proposed by the FDA, and stresses that NAMs should be reviewed or certified before they are used to replace specific animal tests. They list key milestones required to actually achieve the goal of reduced animal testing.

For risk assessment in the context of NAMs, the in vitro to in vivo extrapolation (IVIVE) is a relevant approach. This describes the translation of properties of substances based on in vitro assays to their bioactivity in vivo. A current review of IVIVE with a literature search and explanations on the application areas is given by Han et al. (2024).

For the IVIVE, machine learning (ML) models can be used to translate in vitro-based properties (‘features’) to predictions about the biological properties of compounds. In the context of toxicity prediction, it is most common to use supervised models, where the toxicity status of substances is predicted based on a model that is fitted on a dataset with a known outcome. In the following, no linguistic distinction is made between statistical learning methods and machine learning methods, and all are summarized under the term ‘ML’. Typical types of models include logistic regression, support vector machines, tree-based and tree ensemble methods (e.g. random forests), and deep learning approaches such as neural networks. Especially the latter methods suffer from a lack of (mechanistic) interpretability, which led to the development of methods especially aimed at interpreting ML models.

### Tox21 initiative

In 2008, the federal consortium ‘Toxicology in the 21st Century’, short ‘Tox21’, was formed by four federal agencies in the US. The goals of this initiative focus around evaluating the safety of substances that humans are exposed to in daily life, including the identification of biological mechanisms, the selection of substances for testing, and the development of predictive models (Tox21 2023). Already the first published vision paper of this initiative addresses a paradigm shift from animal-based in vivo testing to in vitro assays, thus avoiding animal experiments while at the same time increasing the relevance of test results for humans (Krewski et al. 2010). Via high-throughput screening assays, a vast amount of data was generated for thousands of compounds. After ten years of the Tox21 initiative, a new strategy with specific expanded focus areas was published. See Thomas et al. (2018) for a summary of the successes, lessons learned, and changes of focus within the initiative.

In the context of the Tox21 initiative, a database containing around 10000 chemical samples, screened in several quantitative high-throughput-screening assays at several concentrations, was created. Thus, this database is also called the ’Tox21 10K library’ (Richard et al. 2021).

Generally, for the meaningful training, evaluation and validation of ML models, a certain amount of high-quality data is required. In addition to the previously introduced Tox21 database, there are many databases focusing on specific types of data, see e.g. Petit (2022) for an extensive overview of available databases.

### The role of machine learning in toxicology

A recent review by Kleinstreuer and Hartung (2024) describes the impact of artificial intelligence (AI) on several key areas of toxicology, such as predictive toxicology, data analysis, risk assessment, and mechanistic research. The authors argue that big data, as described by the ‘Five V’ concept (volume, velocity, variety, veracity, value of data), is now available in toxicological research, thus allowing the use of AI technologies. ML is identified as one of the central concepts of AI, with some sub-concepts including supervised and unsupervised learning, transfer learning, reinforcement learning, and deep learning. A detailed timeline of the advances in AI with references to toxicology is given. Among other challenges in applying AI methods to toxicological data, the need for multidisciplinary expertise is emphasized. This requires collaboration between experts from different areas, both from the application-based side and from the data-analysis side. A PubMed search conducted by the authors based on the keywords AI and Toxicology shows an enormous increase in the number of publications dealing with both of these topics in the last few years.

Applications of ML and AI in several toxicological areas are investigated by Lin and Chou (2022). As relevant areas, the authors identify physiologically based pharmacokinetic (PBPK) modelling, quantitative structure-activity relationship (QSAR) modelling, adverse outcome pathway (AOP) analysis, high-throughput screening (HTS), toxicogenomics, big data, and toxicological databases.

- PBPK models quantitatively describe ADME of a substance, based on the interrelationships of the determinants (physiological, biochemical, physicochemical) of the processes in ADME. These models can be used in the context of risk assessment of substances (World Health Organization 2010).
- QSAR is a modelling approach to quantitatively describe biological activity (including ADME(T)) of a substance based on structural properties of the substance. The structural properties are encoded via molecular descriptors (Lin and Chou 2022).
- Adverse outcome pathways (AOP) provide a framework for causally linking a molecular initiating event (MIE), i.e. an interaction between a substance and an organism, with the adverse effect on individual or population level, via a series of key events (Allen et al. 2014). In the context of the AOP Knowledge Base, a project launched by the Organisation for Economic Co-operation and Development (OECD), a Wiki is provided that contains the current knowledge on AOPs and key events (https://aopwiki.org/).
- HTS means simultaneously testing several thousands of samples for biologically relevant endpoints, making use of robotic equipment. Quantitative HTS describes the generation of concentration-response data in the context of HTS (Shockley 2015).
- Toxicogenomics describes the understanding of toxicological effects of substances or other exposures based on measured gene expression (transcriptomics) or protein values, or other measurements of molecular features (Meier et al. 2025).

For each of the areas, specific applications and examples are given in Lin and Chou (2022). The authors even describe a ‘paradigm shift’ in the framework of risk assessment. While this used to be mainly animal-based, the IVIVE can now be achieved by combining in vitro assays with in silico modelling. However, Lin and Chou (2022) identify six major challenges that need to be addressed, namely the choice of an appropriate ML algorithm, the lack of mechanistical interpretability, the danger of overfitting, the current restriction on yes/no prediction instead of continuous modelling, the overwhelming volume of potentially low-quality data, and the limitations regarding reproducibility due to data and code not being shared. Overall, they conclude that while the existing applications are very promising, it will take time until industry and regulatory agencies completely adopt the methodologies.

### Machine learning for prediction in toxicology

Jia et al. (2023) emphasize the importance of ML and deep learning (DL) approaches (e.g. neural nets) for toxicity predictions, while they point out disadvantages coming from the black-box nature of these types of models. Their review focuses on different types of interpretation approaches for ML and DL models, based on different feature types in toxicological modelling, e.g. structural data (QSAR), pathway data (AOP), or high-dimensional toxicogenomic data. The interpretation approaches include the usage of interpretable feature data, the usage of special methods for interpretation of models, and the development of models based on knowledge.

In a review by Guo et al. (2023), 82 papers published since 2008 were assessed with respect to the employed ML algorithms for binary prediction in pre-specified areas of toxicology. The review focuses on the types of toxicity, the employed ML algorithms, and model validation methods, both for internal and external validation. Overall, the authors conclude that support vector machines and random forests and other ensemble learning methods have been most commonly used in the considered time span. In particular, they stress the importance of high-quality data and interpretability of ML and DL methods for the application of these methods in the context of toxicity prediction.

Wang et al. (2021) give an overview of the statistical models used in the area of *predictive toxicology*, which they define as a field concerning in silico predictions of in vivo toxicological effects. They summarize typical types of classification models and describe additional aspects to consider, e.g. internal validation. For each of the introduced model types, a literature review was conducted in Google Scholar and Web of Knowledge, with the keywords ‘machine learning’, ‘toxicity’, and the name of the model type. The number of hits for each of the model types is compared for two different time spans. Additional summaries regarding the toxicity endpoint, values of different performance measures, types of validation, and sizes of the data set are given. Model performance is compared across the different types of models, stratified by the size of the data set. Finally, the authors conclude that machine learning will be used more and more frequently for predicting toxicity. They identify the quality and quantity of available data as the main bottleneck, and they also emphasize the important role of regulatory acceptance of such methods.

### Interpretability and data quality

The necessity of having ML models tailored to the specific demands of different types of toxicity endpoints is described by Cavasotto and Scardino (2022). In their review, the authors describe the respective current advances and latest developed ML models for the most typical toxicity endpoints, including cardiotoxicity, hepatotoxicity, and toxicity endpoints as defined by the Tox21 initiative. They identify the lack of interpretability, especially the difficulties in obtaining a mechanistic explanation of the toxic outcome as predicted by the ML model, as one of the main challenges in ML-based toxicity prediction. Still, they see a potential for including ML-based toxicity prediction into the pipeline of drug development, provided that enough high-quality data will be available in the future.

For the robust fitting of ML models, enough high-quality data are required. Vo et al. (2020) identify three types of big data in the toxicity context, namely data curated in big databases, high-throughput-screening, and toxicogenomics data. They summarize the main properties, advantages, and limitations of these data types, and have curated a list of relevant databases.

Sinha et al. (2023) focus explicitly on DL models. They claim that, in contrast to traditional ML models such as regression or random forests, no feature generation is required for DL models since these models can build networks based on raw data. Different types of application areas, including prediction of chemical toxicity, adverse drug reactions, and image-based toxicity prediction, are discussed together with examples of fitted DL models in the respective areas. One of the main challenges identified by the authors is the availability of sufficient data, and they provide an overview of several relevant databases. The authors emphasize that, especially with non-interpretable DL models, ensuring responsible usage of these models is very important, referring specifically to concerns regarding privacy, bias, and fairness.

Hartung (2023a, 2023b) gives a positive prospect on the integration of AI technologies in the area of toxicology, while legal and ethical principles need to be upheld. Ideally, the application of these technologies should always be accompanied by a cross-disciplinary team of human experts. The author states the importance of toxicological research, but at the same time, that the area is typically slow in adapting new technologies and approaches. However, in recent years, a shift in the available data could be observed, in the sense that before, the focus was on small-scale in vivo studies, and now there are increasingly high amounts of data available, e.g. by HTS or omics experiments. Relevant application areas of AI in toxicology are given, among others, by pattern finding in HTS, improved risk prediction, and personalized toxicology. The important issues to address are data availability and quality, and the interpretability of AI models. Furthermore, it is stressed that AI cannot solve issues in bad data quality, such that the processes of generating evidence still need to be improved.

### Our review of current practices and reporting gaps

The comparison of the different types of machine learning models via their performance, as done in the review by Wang et al. (2021), is problematic when comparing different methods, since for a fair comparison, different methods would need to be trained and evaluated on the same dataset. Still, this shows that there is an interest in performing such review studies in the area of toxicology. While reviews are relevant for practitioners to get an overview of the most suitable methods, they are also interesting from a method development point of view, to identify areas for targeted research and guidance.

Thus, in this work, a review is presented that builds on the previous work presented by Lin and Chou (2022) and Wang et al. (2021), but extends it in several ways and has a different focus. Specifically, we performed a keyword-based search in the database PubMed (https://pubmed.ncbi.nlm.nih.gov/) and additionally in the journal *Computational Toxicology* (Comput Toxicol). The keywords were ‘Machine Learning’, ‘Toxicology’, and either of the areas ‘QSAR’, ‘PBPK’, ‘AOP’, ‘Predictive’, and ‘Big Data’, identified by Lin and Chou (2022). We included full-text research articles from the years 2022 to 2024, with the search being conducted in June 2024 and updated in September 2025.

Only papers providing details on ML models for binary toxicity prediction were included. We decided to limit ourselves to binary prediction models because this is a very relevant class of models in the context of toxicology. Another class of models would be given by regression models, i.e. models predicting a continuous / quantitative endpoint. In situations where the toxicity of a compound is evaluated depending on the administered concentration or dose, possible endpoints include alert concentrations or doses, such as minimal effective doses or effective concentrations (Kappenberg et al. 2023). While many of the assessed items of our review are equal for classification and regression models, there are differences e.g. in relevant hyperparameters and performance measures between the two types of models. In addition, there are some models that are only suitable for binary classification, but not for regression, or vice versa. Since we wanted to compare the aspects of our review across all assessed records in a meaningful way, we limit the review to classification models.

For basically all papers, the development of a predictive model was the main aspect of the publication. For the 96 papers finally included in the review, numerical aspects (size of the dataset, number of models, number of features) were collected, alongside information on the fitted model, including hyperparameter optimization, the usage of ML interpretation methods, information on internal and external validation, the usage of performance measures, the handling of missing values, and some aspects regarding the reporting of the results.

This review gives an overview of the most used methods, potential gaps in the application of validation and interpretation techniques, and issues in the reporting of results. The review does not aim to make any statements regarding the suitability of the different ML methods for different types of prediction tasks, or to comment on specific performances of methods. Rather, it can be used as a starting point for tailored methodological research and guidance.

Our review differs from the reviews presented by Wang et al. (2021) and Guo et al. (2023) in that the focus is not on the comparison of the respective models in terms of their performance measures, but instead on the information on choice of models, internal and external validation, hyperparameter tuning, and the selected performance measures. In addition, the time frame for the included papers is restricted to a shorter, more recent time. Thus, more aspects are considered, but partly analysed on a high-level basis to get an overview of the field, without going too deep into single publications. The work by Lin and Chou (2022) is example-based, while our review provides a comprehensive overview of all published analyses fitting the inclusion criteria.

The Preferred Reporting Items for Systematic Reviews and Meta-Analyses (PRISMA) statement is a 27-item checklist, in addition to a 12-item checklist specifically for the abstract, to provide guidance in the reporting of a systematic review (Page et al. 2021). The review conducted here is not a systematic review in the sense that studies with a certain intervention are analysed, possibly via meta-analysis, concerning a certain outcome. Instead, a broad overview of the different areas of application of machine learning methods in toxicological research is given, without a detailed synthesis of outcomes. Still, the PRISMA checklist offers valuable support in planning, conducting, and reporting the results of reviews, such that the fitting items of the checklist were followed as closely as possible.

This review is structured as follows. The outline and the specific search strategy of the review are explained, with a detailed presentation of all items that are surveyed. An overview of the reviewed papers is given, and the results of the review are presented, considering all previously introduced review items. Finally, a conclusion regarding the main findings of the review, and an outlook on potential further research directions is given.

## Literature review

In this section, the procedure for the literature review is explained. First, the search strategy, including all sources and exclusion criteria in the screening step, is described. Then, all considered (review) items and their relevance within the fitting of ML models are briefly introduced, and where applicable, some additional details are given.

### Search strategy

The main basis for the literature review about binary prediction models in the context of toxicology presented in this work is the PubMed database (https://pubmed.ncbi.nlm.nih.gov/). This database is maintained by the United States National Library of Medicine (National Institutes of Health) and includes more than 39 million citations for biomedical literature (as of September 2025). In addition, the journal *Computational Toxicology* (Comput Toxicol) was considered. This journal is not indexed in the PubMed database, but it is highly relevant for the task of this literature review, since its scope matches the scope of this review very well.

Both sources were searched with the same combination of keywords. These are based on the previous works by Lin and Chou (2022) and Wang et al. (2021), and were given by ‘(Machine learning) AND (toxicology) AND (...)’, where (...) is one of the keywords QSAR, PBPK, AOP, Predictive, Big Data. Papers that were found using several of these keywords were not considered as duplicates, but for each paper, the keywords that led to finding that paper were saved. For the PubMed database, only full-text articles were considered, and no preprints, and for the journal Comput Toxicol, only research articles were considered. Papers published in the years 2022, 2023, and 2024 were included. The search was conducted on June 26th, 2024, for both sources and updated on September 12th, 2025, to include the remaining published articles of 2024.

In the screening step, the identified records were assessed for eligibility to be included in the review. The comprehensive list of exclusion criteria is given in Table 1. In the case that more than one exclusion criterion was applicable for any record, the first identified criterion was always noted.

**Table 1:** List of exclusion criteria for the literature review with short explanations

Exclusion criterion	Explanation
AOP model only	Only an AOP model is trained, see the manuscript for further comments on this criterion
Application only	No ML model is fitted, only an existing model is evaluated on data
Multiclass	Classification with more than two classes, e.g. ordinal regression or multinomial regression
Consensus model	Results of several models, possibly published in several papers, are summarised and presented as a new model
Database	A (newly built) database is explained or introduced, without focus on fitting of ML models
Opinion	Opinion-based article, e.g. an Editorial or Letter to the Editor
Errata	The record is an erratum to a previously published paper
Guidance	The record provides guidance on specific steps of fitting ML models
Incomprehensible	The relevant items could not be extracted from the paper, or it was unclear whether the paper should even be included
No ML model	No ML model is trained within the paper
Non-Tox	Non-toxicological application, e.g. a clinical application with only short references to toxicological properties of the considered substances
Overview	The paper gives only an overview of the state of the art or of different types of models and applications
Regression model	A non-binary and non-categorical outcome (e.g. continuous) is predicted
Review	The paper is a (systematic) review of certain aspects of ML models in toxicology
Text mining model	The fitted ML model is (mainly) a text mining model
Unsupervised model	An unsupervised model, e.g. clustering, is used

Note that ‘AOP’ was used both as a keyword for the database search and as an exclusion criterion for records. In a pre-screening of the records assessed for eligibility, we noticed a discrepancy between the types of statistical methods used for the applications in the area of AOP and those used for other application areas. Many of the items considered in our review did not align with the models as fitted for the AOP area. Thus, the training of only an AOP model was added as an exclusion criterion.

Several researchers performed this review. A comprehensive catalogue of the relevant items, together with additional explanations and their respective possible values, was prepared in advance. This catalogue and frequent meetings and discussions served as measures to unify the results and minimize inter-reviewer variability.

### Considered items

#### Accessibility

The first item assessed within the literature review is the accessibility of the study. This includes the intuitive understanding via a graphical summary of the study. Note that the existence of a graphical abstract was not deemed sufficient to fulfill this criterion, but rather, a dedicated (sub-)figure comprising the main steps of data pre-processing and the fitting and potentially evaluating an ML model must be present. In addition, the availability of data and/or code was assessed. Here, the upload of data either via online supplementary material or data-sharing websites was considered as available data, and similarly, the upload of code via GitHub or other code-sharing websites was considered as available code. The availability of data or code needed to be explicitly formulated in the paper and no further investigation was carried out.

#### Numeric variables

Four aspects summarizing the numeric properties of the records were assessed. These aspects comprise the number of datasets considered within each study, the number of fitted models, the number of samples (*N*), and the number of features (*p*), with the latter assessed both before and after feature selection where possible. However, during the review process, a large heterogeneity between the records regarding the numeric variables became evident, and explicitly quantifying of the number of features was often challenging or even impossible. Examples for difficulties in quantifying the number of features are given by image data, where the specifics of the derived features are unclear, and molecular fingerprints, where the structure of substances is described by high-dimensional vectors (Capecchi et al. 2020). Thus, the collection of numeric variables was restricted to cases where it was unambiguously possible, and the resulting analyses have to be interpreted with caution.

#### Classification methods

One of the main aspects of the review is the assessment of the different types of prediction models. These were grouped into model types as indicated in the following, and for each record, all types of models were extracted. In Table 2, a description or a list of the specific included models is given for each model type.

**Table 2:** List of prediction models and additional information on the specific included models in the respective group, or other comments

Model type	Additional comments or specific included models
Featureless	The predictions are only based on target labels, not on any features
Discriminant Analysis	This includes linear, quadratic, multiple, and regularized discriminant analysis
Linear Model	A linear model is fitted, then a cutoff is used to classify into two groups
Logistic Regression	This includes all kinds of penalized logistic regressions, as well as logistic GAMs (generalized additive models)
SVM	Support vector machines
k-NN	k-nearest neighbours, this includes variants and generalizations, e.g. the K-Star algorithm
Tree-based	This includes all kinds of decision tree methods, random trees, Chi-square automatic interaction detection (CHAID), conditional inference trees, recursive partitioning, etc
Random Forest	In addition to random forests, this includes all kinds of tree ensemble methods, such as extremely randomized trees
Bagging	The concept of bagging requires some base learners, whose specific forms were not assessed here. Note that in principle, random forests are a bagging approach as well, but were included as a separate model type due to their frequent use. The general type ‘Bagging’ was used in cases of other or unspecified base learners
Boosting	This includes methods as XGBoost and AdaBoost. Regarding the base learners, the same as for Bagging holds
Stacking	Regarding the base learners, the same as for Bagging holds
Neural Net	This includes artificial, convolutional, and probabilistic neural nets, multilayer perception models, etc
Bayesian Network	Probabilistic graphical model that represents conditional dependencies, including a naive Bayes classifier, conditional probability tables, and directed acyclic graphs representing Bayesian networks
Large Language Model	Classification of text into two classes with machine learning
Gaussian Process	Prediction based on multivariate Gaussian distributions, also called Gaussian Process Regression
AutoML	Automated Machine Learning, i.e. all steps within the model building are automatically carried out by specific algorithms

#### Interpretation methods

A particular challenge with many ML models is that they are not directly interpretable. Even the rather intuitive method of random forests does not allow a direct assessment of the splitpoints of the individual underlying trees, and thus does not give an easy-to-follow classification rule. The interpretation becomes even more unclear for deep learning models, such as neural nets, working with hidden layers. Thus, fitted models should also be analysed with respect to ML interpretation methods. This aspect was assessed within our review, with the three possible outcomes ‘feature attribution’, ‘feature importance’, and ‘plot-based methods’.

The group ‘feature attribution’ contains Shapley additive explanations (SHAP), local interpretable model-agnostic explanations (LIME), and gradient-based feature attribution. In the LIME approach, a simple model is determined that locally approximates the complex ML model. SHAP values, defined as the average of the contribution across all permutations of a feature set, can be considered as an extension of this approach, see Rodríguez-Pérez and Bajorath (2020) for details.

For the ‘feature importance’, permutation importance and mean decrease in impurity or accuracy are considered. Permutation importance is assessed by comparing the performance of the model on original data and on data where the values of one feature are randomly permuted and thus no longer associated with the outcome. Mean decrease in impurity is a feature importance method, especially for random forests.

The identified ‘plot-based methods’ are the Grad-Cam algorithm (Selvaraju et al. 2020), a graphic visualization of the decision boundary in the context of a fitted SVM, and contribution maps.

#### Hyperparameter tuning

Many classification methods have one or several hyperparameters controlling the behavior of the respective method, which is why these hyperparameters need to be optimized (Bischl et al. 2023). Examples for such hyperparameters are the number of individual trees fitted within a random forest classifier, the cost of margin violations for support vector machines, or the number of layers in neural networks. In our review, we assessed whether the aspect of hyperparameter tuning was mentioned at all. In records where this was the case, we additionally checked whether detailed information on the set of hyperparameters chosen for optimization was given, and whether information on the search strategy (e.g. grid search, random search) was available.

#### Validation

We collected information on which method was used to ensure internal validity. If more than one method was employed, all methods were recorded. The possible classes of methods contain the following:

- A single or repeated train/test-split, which includes cases where a train/validation-split was performed
- Cross-validation, i.e. repeated train-test splits such that each datapoint is in the test set exactly once and in the training set in the other iterations
- A bootstrap-based approach, where models are fitted on resampled data
- Y-randomization

The latter is a method specifically used for the internal validation of QSAR models. The original model is compared in terms of the coefficient of determination to models determined by the same procedure on a dataset, where the response variable is randomly permuted (Rücker et al. 2007). Note that the bootstrap-based approach here explicitly refers to the formal internal validation procedure. ML approaches, such as random forests and bagging, that internally use a bootstrap resampling are not automatically considered to perform bootstrap-based internal validation.

Moreover, it was assessed whether an external validation on an additional, independent dataset was performed.

#### Missing values

One additional aspect considered in our review was how missing data was handled. One option is ‘no missing values’, in which case this only needs to be reported, but nothing needs to be done. In ‘complete case’ analyses, all observations with missing values in one or several variables are omitted, and in ‘selected case omission’, observations with specific missing variables or with more than a certain percentage of missing values are omitted. In the case of ‘variable omission’, variables with missing values are omitted. ‘Imputation’ refers to a method where missing values of one observation are replaced with values based on the other observations, and in the ‘indicator/dummy’ approach, missing values are set to a fixed value, and an additional dummy variable indicates that these values were missing (Groenwold et al. 2012).

#### Performance measures

For the evaluation of binary prediction models, three categories of performance measures are considered.

- The ‘classification’ category contains measures such as sensitivity, specificity, accuracy, positive or negative predictive values, F$_1$-score, and Matthews correlation coefficient.
- The ‘discrimination’ category comprises the area under the receiver-operator characteristic curve (AUC), the C-statistic, sometimes called C-index, and the Gini index.
- The third category is given by ‘calibration’, which entails both graphical approaches and test approaches, e.g. the Hosmer-Lemeshow test.

For each of these categories, it is only assessed whether at least one of the corresponding measures was stated as the result of the binary classification model.

## Results

In this section, the results of the literature review are presented. A brief overview of the record selection is given, followed by a descriptive analysis of the characteristics of the identified records. The main part is the analysis of all previously introduced items considered in this review.

### Study selection

**Fig. 1:**
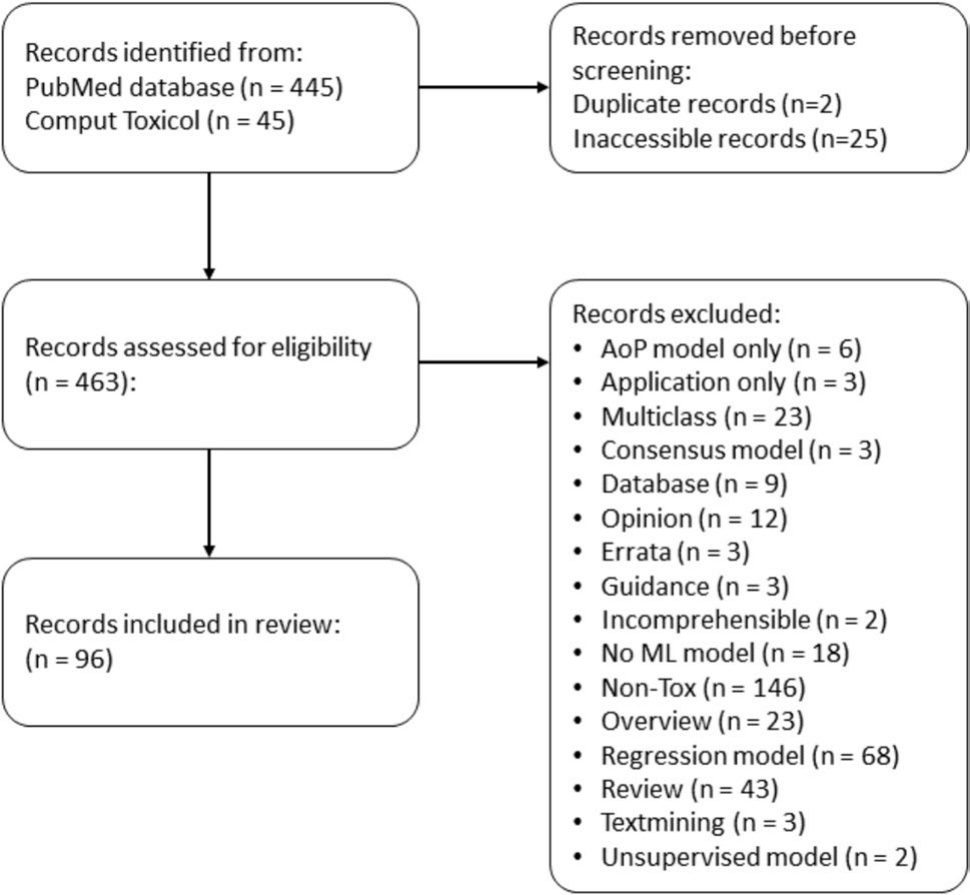
PRISMA flowchart for the study selection, including the number of identified records from the two sources, the number of removed records, and the number of excluded records together with the respective exclusion criteria

With the search strategy as explained before, 445 records were identified in the PubMed database, and 45 records in the journal Computational Toxicology (Comput Toxicol). Out of these, 25 records were not accessible in full text. Even though Comput Toxicol is not indexed as a journal in the PubMed database, it is possible to add individual papers to the database, which makes duplicates possible in this search. Two records were identified both via the PubMed and the Comput Toxicol search. The remaining 463 records were assessed for eligibility according to the pre-specified exclusion criteria (see Table 1). In the case of several reasons for exclusion, the criterion that was first identified as not being fulfilled was always recorded. After screening, 96 records remained in the review. Figure 1 provides the PRISMA flowchart, summarizing the study selection and screening process, together with an overview of the exclusion criteria and their respective number of occurrences.

### Descriptive analysis

A short overview of the descriptive characteristics of the 96 identified records is given in this section. First, the number of records by year and source is given in Table 3.

**Table 3:** Number of identified records by source (PubMed or Comput Toxicol) and year (2022 – 2024)

Source	2022	2023	2024
PubMed	18	28	37
Comput Toxicol	6	4	3

Overall, the 96 records were published in 49 different journals. Table 4 gives a summary of the journal frequency counts. The journal with 13 publications included in the review is *Computational Toxicology*. The two journals with 8 and 7 publications each included in the review are *Chemical Research Toxicology* and *Toxicology*, respectively.

**Table 4:** Summary table of journal frequency counts for the 96 records, published in 49 journals

Frequencies	1	2	3	4	7	8	13
Number of journals	31	10	3	2	1	1	1

The search was conducted with five keywords (QSAR, PBPK, AOP, Predictive, Big Data). Table 5 summarizes the number of records which were flagged for each of the five keywords. Here, it can be seen that the selection of records is driven mainly by the keyword ‘Predictive’, which is relevant for all records except two, and the keyword ‘QSAR’, which is relevant for 36 out of 96 records. The keywords ‘PBPK’, ‘AOP’, and ‘Big Data’ were only seldom (1 – 4 times) relevant.

**Table 5:** Number of times each of the five keywords was relevant for the 96 identified records

Keyword	QSAR	PBPK	AOP	Predictive	Big Data
Yes	36	1	3	94	4
No	60	95	93	2	92

### Results of the items

#### Accessibility

**Fig. 2:**
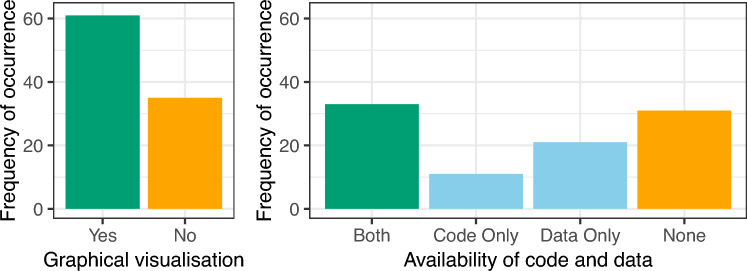
Information about the accessibility of the information presented in the respective paper. Left: Is a graphical abstract available, right: Are code and/or data available

The first aspect considered in the review was the accessibility of the information presented in the respective article, specifically whether a graphical summary allows a simple comprehension of the approaches in the article, and if the underlying raw data and/or code used for training the model are available. The results are summarized in Fig. 2.

While the inclusion of a graphical summary of the overall procedure is quite common and occurs in almost two-thirds of all cases, there are still many papers without any data or code available, or only one of these two available.

#### Numeric variables

As numeric variables, the number of different datasets per paper, the number of fitted models, the sample size (*N*), and the number of features (*p*) were assessed. Table 6 summarizes the number of datasets per paper. Most papers consider only one dataset, but some papers also consider several datasets. The by far highest value of 85 datasets is observed in a paper where the individual datasets are obtained by considering single genes.

**Table 6:** Number of different datasets analysed in each of 96 assessed record

No. of datasets	1	2	3	4	5	6	7	12	16	18	23	85	unclear (many)
No. of papers	59	13	8	2	2	4	1	1	1	2	1	1	1

The number of fitted models could not be retrieved in four cases. For the other 92 records, the number varies between 1 and 3420, with the majority of values in the range from 1 to 72. The complete numbers are summarized in Suppl. Table A.1.

The number of samples (*N*) across the different papers is too heterogeneous for a summary that is intuitive to grasp. In papers with several datasets, the numbers of samples often differ, sometimes substantially, between those datasets, and overall, the numbers range from 6 to close to 800,000.

The number of features (*p*) is even more challenging to assess, for several reasons. Sometimes, only numbers before or only numbers after feature selection are given. In the case of feature selection, the number of features might vary in different analyses. Moreover, the number of features might vary between datasets, and assessing the number of features for data types like molecular fingerprints or image data can be challenging. Thus, both very low feature counts, i.e. in the one-digit range as well as very high counts, reaching into the thousands for image data, are observed.

#### Classification methods

**Fig. 3:**
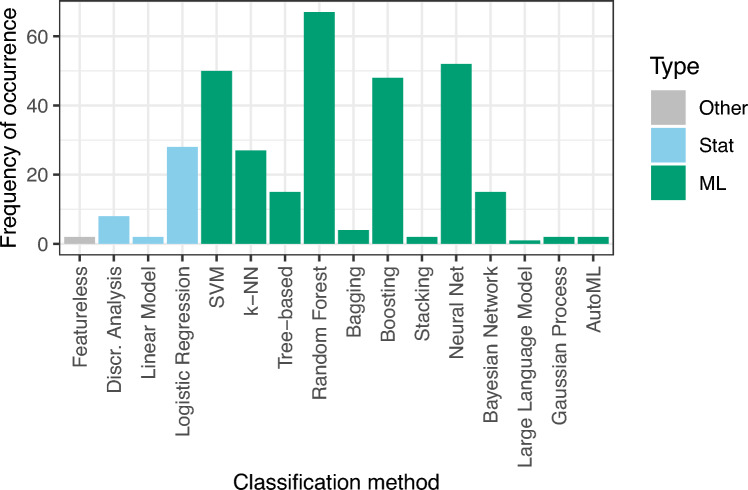
Number of papers in which each classification method was used. The colors indicate a rough differentiation into purely statistical and ML models

To get an overview of the most commonly used methods for toxicological prediction models, for each paper, all applied model types were collected. Figure 3 shows the number of times each model type was used in the 96 identified records. The colors indicate a rough differentiation into purely statistical models and ML models in the widest sense, i.e. not only including deep learning but also statistical learning.

The most commonly used methods (Random Forest, Neural Net, SVM, Boosting) all belong to the area of ML models. These four model types are used in around half of (or, in the case of the Random Forest, clearly more than half of) the 96 identified records.

Often, several model types are fitted within one paper. Table 7 gives an overview of the number of model types (ranging from 1 to 9) fitted to the data in each of the papers. In the majority of papers, 2 to 5 model types are used, with notably many papers with only one type, and a few papers with even more than 5 types.

**Table 7:** Number of model types fitted to the data in each of the 96 identified papers

No. of model types	1	2	3	4	5	6	7	8	9
No. of papers	26	11	12	16	16	7	3	4	1

Note that the number of model types and the number of actually fitted models might differ, since the fitting of two versions of the same model type (e.g. L1 and L2 penalized logistic regression, or artificial and probabilistic neural nets) was only counted as one type (here: logistic regression and neural net, respectively).

#### Interpretation methods

**Fig. 4:**
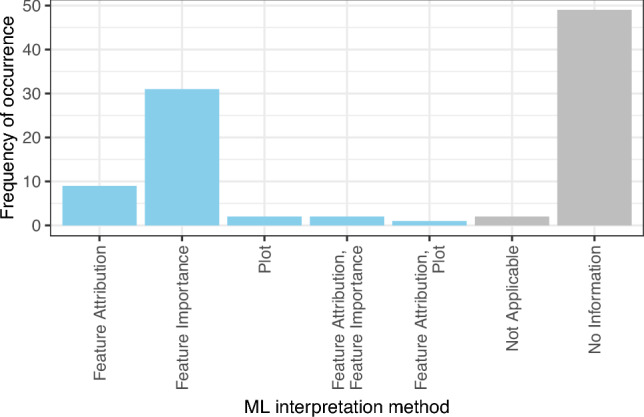
Overview of the interpretation methods. Note that this plot is set up by record, not by occurrence of the individual method

The summary of usage of ML interpretation methods is given in Fig. 4. This summary is created according to the number of assessed papers, i.e. the frequencies in the plot sum up to 96. In more than half of the records, no usage of an ML interpretation method is explicitly indicated. The two cases where ML interpretation methods are not applicable are records where only directly interpretable model types, e.g. logistic regression models, are fitted to the data. Feature importance approaches are more common than feature attribution measures, and the combination of both is only used in two records. Plot-based approaches are only used in three records, and in one of these cases in combination with a feature attribution approach.

#### Hyperparameter tuning

**Fig. 5:**
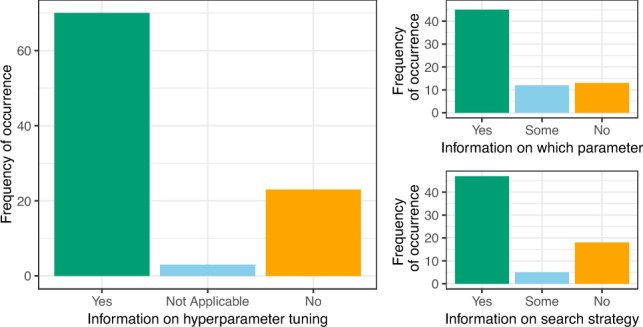
Information on the reporting of hyperparameter tuning, regarding whether there is information about hyperparameter tuning at all (left), about which parameter is tuned (top right), and about the search strategy (bottom right)

Many of the employed prediction models contain hyperparameters that need to be tuned during the fitting of an optimal model. Figure 5 shows the frequency of occurrence of general information on the hyperparameter tuning within the papers, as well as the occurrence of detailed information on the parameter chosen for tuning, and the employed search strategy.

General information is available in 70 of 96 cases. The answer ‘not applicable’ corresponds to papers where only models without any hyperparameters, e.g. unpenalized logistic regression or AutoML, are used for prediction. Still, in more than 20 papers, no information is reported at all. Looking in detail at the 70 papers with available information on hyperparameter tuning, approximately 45 papers have detailed information on the (sets of) hyperparameters in the tuning and on the employed search strategy, respectively. Incomplete, but some information about the parameter in tuning and the search strategy is given for 12 and 5 records, respectively. Still, there are a notable number of papers with no detailed information at all.

#### Validation

**Fig. 6:**
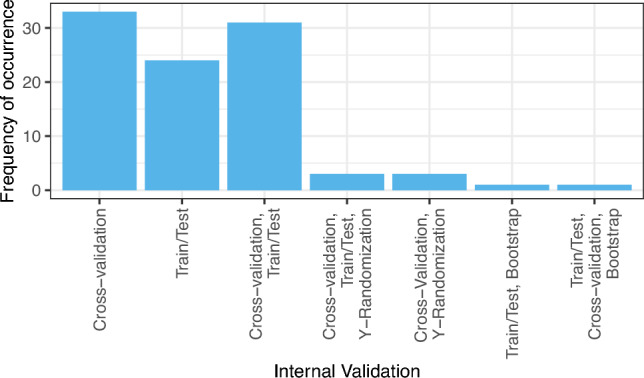
Overview of the employed (combination of) internal validation methods, assessed for each record individually

In all 96 assessed records, some kind of internal validation was performed. Figure 6 shows all combinations of internal validation methods found in the assessed records. In the majority of cases, cross-validation, a train/test-split, or a combination of both was employed. Y-Randomization and Bootstrapping were seldom used, and only in combination with other methods.

External validation on an independent new dataset was performed in only 32 of 96 records. Since information on external validity of a model is crucial to assess its generalizability to other but related datasets, omitting this information means that the application of the model to other datasets may yield potentially unreliable results (Steyerberg 2019). However it could be the case that external validation is performed in follow-up publications, e.g. in comparison to other binary prediction models, which would have been missed in our review.

#### Missing values

**Fig. 7:**
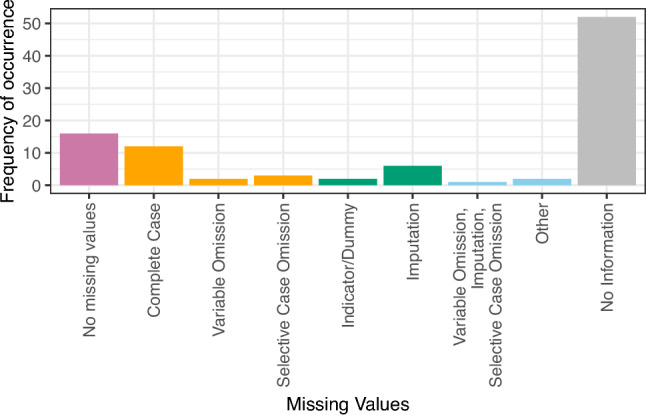
Information on dealing with missing values in the 96 records. The colors give an impression whether omission took place (orange), imputation or dummy coding took place (green), some other approach was taken (blue), or no missing values were in the data (rose), or no information was explicitly stated in the record (grey)

Fig. 7 displays the approaches for dealing with missing values. The most striking observation is that in more than half of the assessed records, no clear information about missing values could be retrieved at all. In many cases, no values were missing in the data. For the remaining records, the complete case analysis and imputation approaches were used most commonly.

#### Performance measures

The performance of prediction models can be assessed via different measures, grouped into the categories classification, discrimination, and calibration. Table 8 displays the number of records in which a performance measure from each of the three classes is employed. The classification performance is assessed in almost all records, discrimination in the majority, and calibration almost never. Additional analyses show that at least one of the two categories, classification and discrimination, is assessed in all records.

**Table 8:** Number of records using a classification, a discrimination, or a calibration performance measure, respectively

	Classification	Discrimination	Calibration
Used	92	69	3
Not used	4	27	93

## Conclusion and discussion

Artificial intelligence (AI) has advanced to play a huge role in many aspects of research. Toxicological research is no exception to this. It is becoming an increasingly data-rich discipline with shifts from small-sample in vivo experiments to large-scale high-throughput screening, and with experiments focusing on high-dimensional omics data. Many recent publications give comprehensive overviews of application areas that profit from the combination of AI and toxicology, such as predictive toxicology, QSAR analyses, and pattern identification in big data experiments. Several machine learning (ML) approaches, such as tree-based methods or different kinds of (deep) neural networks, are popular methods for the prediction of relevant toxicity endpoints. The choice of methods needs to be tailored to the type of toxicological endpoint and to the specific toxicological question. One of the most crucial aspects when applying AI/ML to toxicological data is the availability of sufficient high-quality data. Consequently, researchers can benefit from numerous large databases with a focus on different types of toxicological data that have emerged in recent years.

However, there are also critical disadvantages of AI/ML. A major aspect is typically the lack of direct interpretability of ML models, i.e. they are ‘black box’-type models. There exist several approaches to combine expert knowledge with ML models, or to perform post-hoc analyses of individual features in a model to allow their interpretation. When fitting an ML model on an entire dataset and reporting the performance without taking care of internal validation approaches such as cross-validation, ML models always come with the danger of severely overfitting the given data and thus not performing well on new, independent data. Especially due to the complex nature of ML models, precise reporting of the performed steps in data preparation and model fitting is therefore of utmost importance.

To assess the current state of the art in the application of ML models, we have performed a literature review on published papers including a binary prediction model, indexed in PubMed or published in the journal ‘Computational Toxicology’ in the years 2022 to 2024. Records were identified using a keyword-based search with the terms ‘Machine Learning’ AND ‘Toxicology’ AND (...), where (...) is one of the relevant areas QSAR, PBPK, AOP, Predictive, Big Data. Several exclusion critera were defined, and in the end, 96 records were included in the review and assessed for the accessibility of code and data, information about number of datasets, number of fitted models, sample sizes, number of features, the used classification method, information on interpretation methods, hyperparameter tuning and internal and external validation, the dealing with missing values, and the reporting of performance measures.

The main observations are insightful and summarized in the following. The numerical variables, especially the number of features and the sample sizes, are often hard to quantify and summarize. Methods might also differ in the step where feature selection is performed, either before the model is fitted (e.g. via filter criteria), or within the fitting of the model, as seen e.g. in a LASSO-type penalized regression, which makes comparisons harder. In addition, with image-based features, a clear quantification of these is often not even possible.

With regard to the chosen methods, random forests, neural nets, and SVMs are most popular. A frequent lack of reporting of the hyperparameter tuning and the handling of missing values could be observed, in the sense that this information is not always fully provided in a manner that the analysis could be reproduced. Only in about a third of all assessed records, both data and code are provided, and in an additional third of the cases, only one of these two.

With respect to performance measures, always, measures from at least one of the two categories, classification and discrimination, are calculated. However, calibration measures are almost never considered.

The key elements required for comprehensive reporting of binary classification models in toxicology are as follows:

- Dataset characteristics
  - Number of samples (*N*)
  - Number of candidate features (*p*, before selection or regularization approaches)
  - Number of selected features
- Model development
  - Type of classification model
  - Hyperparameter tuning (set of parameters used for tuning, tuning strategy, results)
  - Feature selection (either as explicit approach, e.g. best subset selection, or achieved via regularization approaches, e.g. LASSO)
  - Interpretation
- Data quality
  - Presence of missing values
  - Handling missing data
  - Further data cleaning and/or normalization
- Validation
  - Internal validation approach
  - External validation (including details on the dataset used for external validation)
- Model performance
  - Performance measures for classification, discrimination, calibration
- Reproducibility and accessibility
  - Visualization of the procedure
  - Availability of code
  - Availability of data

These elements are based on the items examined in our review. Note that this list does not include any relevant toxicological aspects, such as the type of toxicity (e.g., cardiotoxicity, hepatotoxicity) assessed and the endpoint of interest, but focuses on the specific aspects of data analysis in the broad sense.

In our review, far fewer papers (96 out of 463) were included than initially expected. This was mostly due to papers having a clinical focus, but still coming up in the keyword-based search, because toxicological aspects of compounds were also discussed in the paper. Initially, within the review, an idea was to additionally assess the authors’ backgrounds and find out whether the team was made up of interdisciplinary experts from both the toxicology and the statistics/computer science area. However, this information can most often not be retrieved based solely on the affiliation, and performing in-depth analyses for all authors of the oftentimes large teams of authors on one single publication was not feasible.

Overall, this work serves as an initial step toward recognizing the importance of providing tailored guidance and software solutions for performing binary classification tasks in relevant areas of toxicology, making use of ML models.

## Supplementary Information

Below is the link to the electronic supplementary material.

Supplementary Material 1.

## Data Availability

No new data were created or analyzed in this study.
