# Supplementary material for: A review of machine learning in toxicology: current practices and reporting gaps
Source: Arch Toxicol. 2026 Apr 30;100(8):3491–505. doi: 10.1007/s00204-026-04393-0 (PMC13379476; doi:10.1007/s00204-026-04393-0)
Supplement: Supplementary file 1 — Supplementary Material 1. [file 204_2026_4393_MOESM1_ESM.pdf]

Supporting Information for 'A Review of Machine Learning in Toxicology: Current Practices and Reporting Gaps'

No. of models	1	2	3	4	5	6	7	8	9	10	12	15	16
No. of papers	9	8	6	1	12	9	4	1	4	3	2	1	2
No. of models	17	18	20	24	25	27	28	35	44	45	48	53	58
No. of papers	1	1	3	1	1	1	1	1	1	1	5	1	1
No. of models	60	66	68	69	72	100	103	144	306	765	3420	NA	
No. of papers	1	1	1	1	1	1	1	1	1	1	1	4	

Suppl. Table A.1: Number of fitted models per paper. Note that the value 100 here is an approximation, in the respective record it is stated that 'near 100' models were fitted.
